# Does RV fibrosis have an effect on mortality in patients hypertrophic cardiomyopathy? An LV vs. RV national death index (NDI) study

**DOI:** 10.1186/1532-429X-17-S1-P179

**Published:** 2015-02-03

**Authors:** Robert W Biederman, Nessim N Amin, Sirikarn Napan, Diane V Thompson, Moneal Shah, Mark Doyle

**Affiliations:** Cardiac MRI, Allegheny General Hospital, Pittsburgh, PA USA; Neurology, Cleveland Clinic, Cleveland, OH USA

## Background

### Introduction

CMR has become the leading technique to define the clinical impact of hypertrophic cardiomyopathy (HCM) providing complete coverage of both ventricles with high spatial resolution. Gadolinium delayed-enhancement (DHE) accurately identifies regions of myocardial fibrosis. Via CMR, innumerable studies have established that LVH is the predominant phenotypic expression of HCM. It is well known that myocardial fibrosis can occur in patients with HCM and is independently linked to a poorer prognosis than those without fibrosis by CMR.

### Hypothesis

We hypothesize that there if there is significant RV involvement in HCM when incorporating a CMR analysis for RV hypertrophy and fibrosis this will have an independent effect on mortality.

## Methods

Review of all patients referred for HCM was performed. SSFP/DHE was used to diagnose patients with HCM, using gadolinium administration (0.15mmol/kg, MultiHance, Bracco Diagnostics, Princeton, NJ). Post-injection (2/10 minutes) DHE images were obtained using manual T1-weighted, inversion recovery preparations. Regions of myocardium with abnormally high signals were designated as fibrotic. LV/RV mass indices (LVMI/RVMI) and ejection fractions were calculated. Fibrosis was semi-quantitatively assessed in both ventricles. All patients will undergo National Death Index search for mortality.

## Results

Via 72 patients referred for HCM from 2007-2013, 47(65.27 %) were CMR confirmed. The mean LVMI was 108 ±44gm^2^ while the mean RVMI was 30±21 gm^2^. All patients met formal LVH criteria while 34/47 (72%) met RVH criteria. Out of the RVH positive patients, 26/34(76%) patients were LV LGE positive and 18/34 (52%) were RV LGE positive. 34/47(72%) had evidence of LV fibrosis while 24/47 (51%) had evidence for RV fibrosis. Only 2 patients with RV fibrosis had absent LV fibrosis. There is a negative correlation between LVH (mean 108 ±44gm^2^) and LVEF (67% ±10%) of -0.097. Three of 72 patients died of CV deaths as per NDI with all 3 having LV LGE of which 2 had RV LGE (p=NS). Neither the degree of RV hypertrophy nor the amount of RV LGE was shown to be predictive of death for the 2 pts with RV involvement (p=NS).

## Conclusions

The frequency of RVH/fibrosis in the setting of HCM is common especially with the knowledge that this phenomenon is rarely described. Nevertheless, given the myriad of genetic abnormalities, there is no reason to expect the phenotypic expression *should* be limited to the LV. However, neither the impact of RV hypertrophy nor presence of RV LGE has an independent impact on mortality.

## Funding

Internal.

